# Complete chloroplast genome sequence of *Thalictrum viscosum* W.T.Wang & S.H.Wang, 1979 (Ranunculaceae)

**DOI:** 10.1080/23802359.2022.2113751

**Published:** 2022-09-05

**Authors:** Haohong Cai, Wenbo Shi, Song Weicai, Weiqi Han, Shuo Wang

**Affiliations:** College of Marine Science and Biological Engineering, Qingdao University of Science and Technology, Qingdao, China

**Keywords:** *Thalictrum viscosum*, Ranunculaceae, chloroplast genome, phylogenetic relationship

## Abstract

*Thalictrum viscosum* W.T.Wang & S.H.Wang, 1979 is a flowering plant species in family Ranunculaceae that is endemic to Yunnan province of China. To facilitate genetic study of *T. viscosum*, we *de novo* assembled and annotated the complete chloroplast (cp) genome of *T. viscosum* for the first time. The total length of the cp genome of *T. viscosum* was 155,984 bp, with a GC content of 38.4%. The *T. viscosum* cp genome had a typical quadripartite structure with a large single-copy region of 85,339 bp, a small single-copy region of 17,656 bp, and a pair of inverted repeat regions of 26,495 bp. The cp genome consisted of 133 genes, including 87 protein-coding genes, 38 transfer RNA genes, and eight ribosomal RNA genes. We performed phylogenetic analysis of *T. viscosum* with the maximum-likelihood phylogenetic tree and indicated that *T. viscosum* was closely related to *T. cirrhosum* and *T. foeniculaceum.*

*Thalictrum viscosum* W.T.Wang & S.H.Wang, 1979 is a flowering plant species in family Ranunculaceae. It grows in grassy places of the valleys or riversides and is endemic to Yunnan province of China (Zeng et al. 2020). Besides, the *T. viscosum*, as a medicinal herb with valuable botanical components that has received much attention (Chen et al. 2003; Khamidullina et al. 2006; Sharma et al. 2020). The chloroplast (cp) genome is a useful genetic tool for conservation research of endangered species (Arias et al. 2020; Song et al. 2022), but so far, there are no reports on the cp genome of *T. viscosum*. To facilitate further research on *T. viscosum*, we assembled and annotated the complete cp genome of *T. viscosum* for the first time. In addition, we performed phylogenetic analysis of *T. viscosum* to identify its phylogenetic relationships with other Ranunculaceae species.

The plant material of *T. viscosum* was collected in Kunming City, Yunnan Province, China (25°04′N, 102°42′E). We obtained approval for this research both from the local government and from the Kunming Institute of Botany, Chinese Academy of Sciences. A specimen was deposited at Qingdao University of Science and Technology (Chao Shi, chsh1111@aliyun.com) under the voucher number TV202223. We washed the plant material with distilled water. Then, we extracted cpDNA from roughly 20 g of fresh plant leaves of *T. viscosum* using the more effective and improved high salt method (Shi et al. 2012). Subsequently, DNA quality was determined by spectrophotometric assessment. The high-quality DNA of *T. viscosum* was used to generate a genomic library for sequencing in Novogene Company (Beijing, China) with Illumina Hiseq 4000 platform. Low-quality reads and adaptors were clipped with Trimmomatic v0.36 (Bolger et al. 2014) and quality control was assessed by FastQC (Brown et al. 2017). Eventually, about 2.7 Gb of high-quality reads were generated. The complete cp genome of *T. viscosum* was assembled with NOVOPlasty v4.3.1 software (Dierckxsens et al. 2016). The assembly results were aligned by BLASTn (Johnson et al. 2008) to find the reference genome with the highest similarity and finally *T. foeniculaceum* (accession number NC_053570) was chosen as the reference sequence. The GeSeq software (Tillich et al. 2017) was used to annotate the protein-coding genes (PCGs), ribosomal RNA (rRNA) genes, and transfer RNA (tRNA) genes contained in the genome. Lastly, the annotation results were manually corrected against the reference genome by Sequin (Lehwark and Greiner 2019). The assembled *T. viscosum* cp genome sequence was submitted to the GenBank database under accession number MZ442609.

The total length of the *T. viscosum* cp genome was 155,984 bp, with a GC content of 38.4%. The makeup of the nucleotides was asymmetric (30.5% A, 19.6% C, 18.8% G, and 31.1% T). The *T. viscosum* cp genome had a typical quadripartite structure. A large single-copy (LSC) region of 85,339 bp and a small single-copy (SSC) region of 17,656 bp were separated by two equal 26,495 bp inverted repeat (IR) regions, IRa and IRb. The cp genome consisted of 133 genes, including 87 PCGs, 38 tRNA genes, and eight rRNA genes. A total of 23 genes with introns were annotated in the *T. viscosum* cp genome, with two genes (*clpP1* and *ycf3*) having two introns and 21 genes having one intron. The *trnK*-UUU gene contained the largest intron, with a length of 2,521 bp, while the *trnL*-UAA gene contained the smallest intron, with a length of 531 bp. To determine the phylogenetic position of *T. viscosum*, we constructed a phylogenetic tree using the complete cp genomes of 20 Ranunculaceae species that have been released in the NCBI database and by using *Coptis chinensis* as an outgroup (Figure 1). The complete cp genome sequences of 20 Ranunculaceae species were aligned using MAFFT v725 (Katoh and Standley 2013). The Modeltest (Posada and Crandall 1998) was used to determine the best nucleotide substitution model. The best model, GTR-GAMMA (GTR + G), was selected to construct the phylogenetic tree based on the Bayesian information criterion (BIC). The maximum-likelihood (ML) tree was constructed using MEGA-X software (Kumar et al. 2018). The ML phylogenetic tree indicated that *T. viscosum* was closely related to *T. cirrhosum* and *T. foeniculaceum* and support the conclusion of the earlier study (He et al. 2021) that *T. baicalense* was closely related to *T. tenue*, *T. minus*, and *T. petaloideum*.

**Figure 1.**
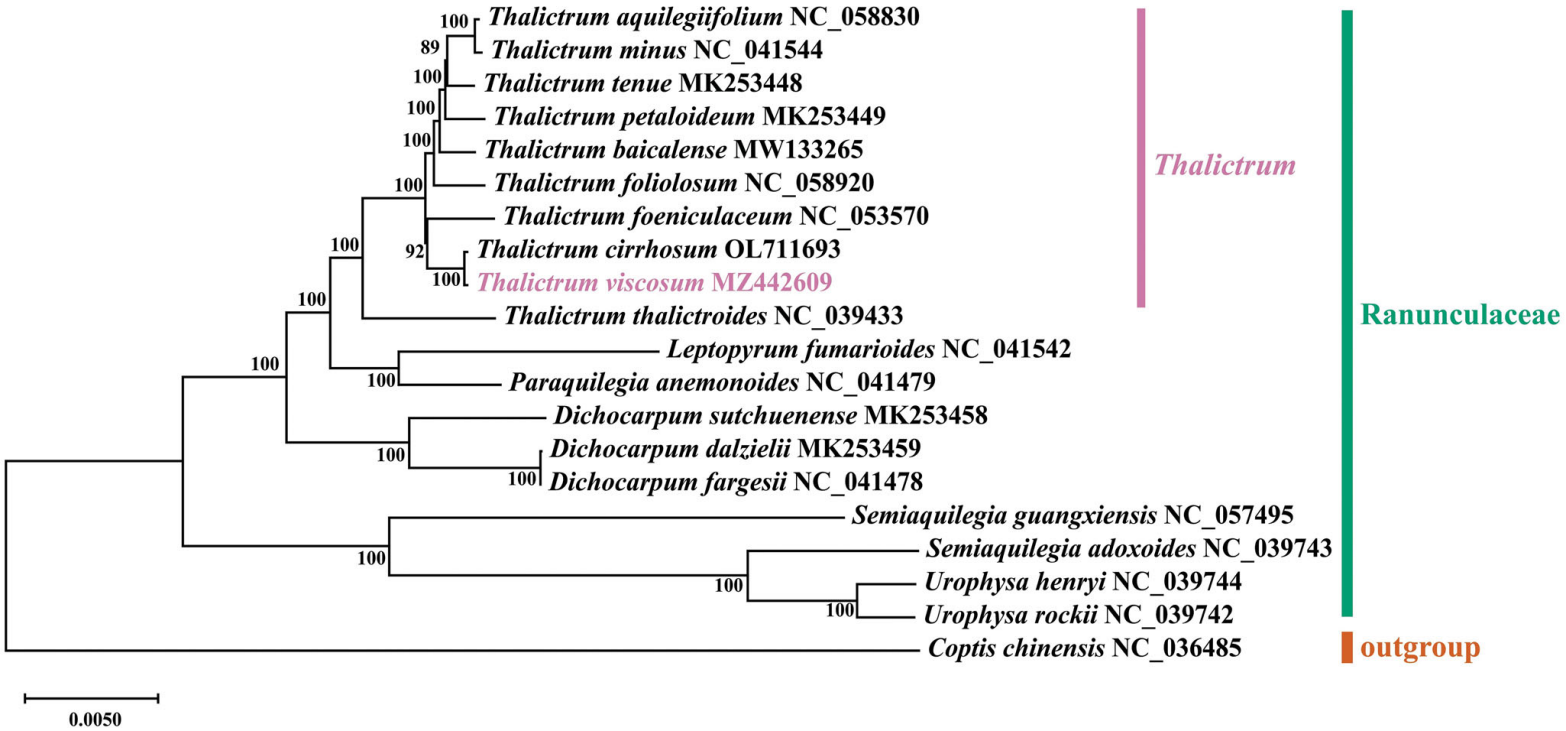
The maximum-likelihood tree indicates the phylogenetic relationships of 20 Ranunculaceae species, with the number on each node representing the bootstrap support value. Following the species is the GenBank accession number of the chloroplast genome sequence.

## Data Availability

The genome sequence data that support the findings of this study are openly available in GenBank of NCBI at https://www.ncbi.nlm.nih.gov/ under the accession MZ442609. The associated BioProject, SRA, and Bio-Sample numbers are PRJNA818965, SRR18441631, and SAMN26892146, respectively.
